# Imaging for Dupuytren disease: a systematic review of the literature

**DOI:** 10.1186/s12891-019-2606-0

**Published:** 2019-05-17

**Authors:** Sanne Molenkamp, Roel J. M. van Straalen, Paul M. N. Werker, Dieuwke C. Broekstra

**Affiliations:** Department of Plastic surgery, University of Groningen, University Medical Center Groningen, BB81 Hanzeplein 1, 9713 GZ Groningen, The Netherlands

**Keywords:** Dupuytren contracture, Ultrasonography, Magnetic resonance imaging, Imaging, Systematic review

## Abstract

**Background:**

As treatment of Dupuytren disease (DD) is expected to shift towards prevention of progression, the use of imaging in patients with DD becomes more important. In this systematic review an overview is given of the different methods for and applications of imaging for DD that have been described.

**Methods:**

The MEDLINE and EMBASE databases were searched for articles reporting the use of imaging in patients with DD, published before May 17, 2018. Studies were systematically examined in two rounds by two observers according to the PRISMA systematic. All studies containing original data on imaging for DD were considered for inclusion.

**Results:**

Three hundred and seven unique studies were identified, of which 23 were included in the study. Only studies on the use of ultrasound (US) and magnetic resonance imaging (MRI) were identified. Broadly, articles could be divided into 5 categories. Seven studies were found on diagnosis, two on measurement of disease extent, four on measurement of disease activity, seven on guidance of minimally invasive procedures and five studies on evaluation of treatment. According to the Oxford CEBM, the levels of evidence were low, ranging from level 3 to 5.

**Conclusions:**

A variety of applications for US and MRI for patients with DD has been described. Based on the results of this review, the largest value for imaging lies in the measurement of disease activity and the follow-up of treatment of patients with early stage disease. Unfortunately, the overall level of evidence of the available literature was low. Future research is necessary to define the exact value of US and MRI in the management of patients with DD.

## Background

Dupuytren disease (DD) is a benign fibromatosis of the palmar fascias of the hand. Much has been speculated about the aetiology of DD and about why some patients have a more aggressive course of the disease than others. Both intrinsic and extrinsic factors, such as age, gender, genetic predisposition, co-morbidity, manual labour and hand-trauma, seem to play a role [1–5]. However, despite the increase in knowledge on risk-factors and predictors for the origin and progression of DD, the disease course remains extremely variable [6, 7]. While some patients require frequent operations to maintain functionality of affected hands, some remain stable after one operation, while others only develop nodules without any relevant complaints. This is why it is essential that in the future, evaluation and treatment of DD should be focused more on the individual, based on genetic predisposition, environmental factors and clinical features [8, 9]. Ideally, an individualized algorithm for DD will enable the differentiation of patients with slow progression and a good prognosis from patients that are at risk of aggressive disease, who have to be monitored closely and treated at the right moment using the most appropriate treatment. Such an algorithm would assist in the selection of the most appropriate treatment, which range from non-invasive (pharmacotherapy, radiotherapy or splint therapy), to minimally invasive (percutaneous needle fasciotomy (PNF) or Collagenase *Clostridium Histolytocum* (CCH) injections) to more invasive (limited fasciectomy or dermatofasciectomy).

Currently, physical examination of the hands is the gold standard for assessment of disease stage, disease extent and disease progression [10]. However, physical examination only gives us at best a two-dimensional idea of the extent of disease. Also, with physical examination disease activity can only be determined by performing follow-up in time. An alternative to measure disease activity, is by what the patient reports. However, the reliability of this method is questionable as it is subjective. Finally, physical examination cannot always give us reliable information about changes in anatomy (e.g. displaced neurovascular bundles). The introduction of imaging for DD could therefore be an important extension to the development of an individualized treatment algorithm and to the improvement of the predictability of results of existing treatment modalities. Ultrasound (US) depicts echogenicity and is well suited to reveal dimensions of a soft tissue lesion in the sagittal and transverse plane. With computed tomography (CT) and magnetic resonance imaging (MRI) three dimensions of soft tissue laesions can be displayed in detail. However, the use of MRI is more common, as there is no additional radiation exposure. All three imaging modalities can give additional information about vascularity, size and location [11–14].

The literature on the use of imaging to facilitate clinical examination and treatment of patients with varying stages of DD, is expanding [15]. However, no overview of the possible applications of imaging for DD is available yet, which is why this systematic review was conducted. The aim was to investigate what applications have been described previously for different imaging modalities and DD, hereby also pointing out the topics that are in need of further research.

## Methods

A systematic literature search was performed on May 17, 2018 to identify articles on the use of ultrasound (US), magnetic resonance imaging (MRI) and/or computed tomography (CT)/positron emission tomography (PET) for patients with DD. The MEDLINE and EMBASE database were searched for relevant articles using the queries reported in Table 1. These queries were created together with an information specialist at our medical library.

**Table 1:** Search strategy per database

Database	Search query
MEDLINE	(“Dupuytren Contracture”[Mesh] OR dupuytren*[tiab] OR palmar fibromatos*[tiab]) AND (“Ultrasonography”[Mesh] OR “ultrasonography” [Subheading] OR “Tomography”[Mesh] OR ultraso*[tiab] OR “radiography” [Subheading] OR echograph*[tiab] OR radiograph*[tiab] OR tomograph*[tiab] OR sonograph*[tiab] OR CT [tiab] OR PET [tiab] OR MRI [tiab] OR imaging [tiab])
EMBASE	(‘dupuytren contracture’/exp. OR dupuytren*:ab,ti OR ‘palmar fibromatosis’:ab,ti) AND (‘echography’/exp. OR ‘radiodiagnosis’/exp. OR ultraso*:ab,ti OR echograph*:ab,ti OR radiograph*:ab,ti OR tomograph*:ab,ti OR sonograph*:ab,ti OR ct:ab,ti OR pet:ab,ti OR mri:ab,ti OR imaging:ab,ti)

Two independent observers (S.M. and R.v.S.) assessed the articles in two assessment rounds. In the first round the titles, abstracts and keywords were screened for the combination of DD and US/MRI/CT/PET. For the full-text round, articles were assessed for the use of imaging for DD. Articles on therapeutic ultrasound, also known as shockwave therapy were excluded. When studies mentioned the use of imaging merely for the investigation of post-operative complications of surgery for DD (e.g. flexor tendon ruptures) and not for the post-operative follow-up of Dupuytren tissue, they were also excluded. As the aim of this study was to generate an overview of the possible applications of imaging for DD, all studies containing original data were considered for inclusion, including case-reports and conference proceedings. The inclusion and exclusion criteria are shown in Table 2. If consensus between the two observers could not be reached, a third observer (D.C.B.) was consulted. All included articles were assessed for level of evidence, using the Oxford CEBM Levels of Evidence [16]. This systematic review was written according to the PRISMA reporting guideline for systematic reviews [17].

**Table 2:** Inclusion and exclusion criteria used in different rounds

Round 1: Title + abstract	Round 2: Full-text
Inclusion criteria	Inclusion criterion
- Patients with DD	Imaging (US/MRI/CT/PET) used for the
- Imaging (US/MRI/CT/PET)	assessment of DD
Exclusion criteria	Exclusion criteria
- Language other than Dutch, English, German or French	- Ultrasonic therapy for DD
- No original data	- Imaging for post-operative complications
	- No original data

*DD* Dupuytren Disease, *US* ultrasound, *MRI* magnetic resonance imaging, *CT* computed tomography, *PET* positron emission tomography

## Results

**Fig. 1:**
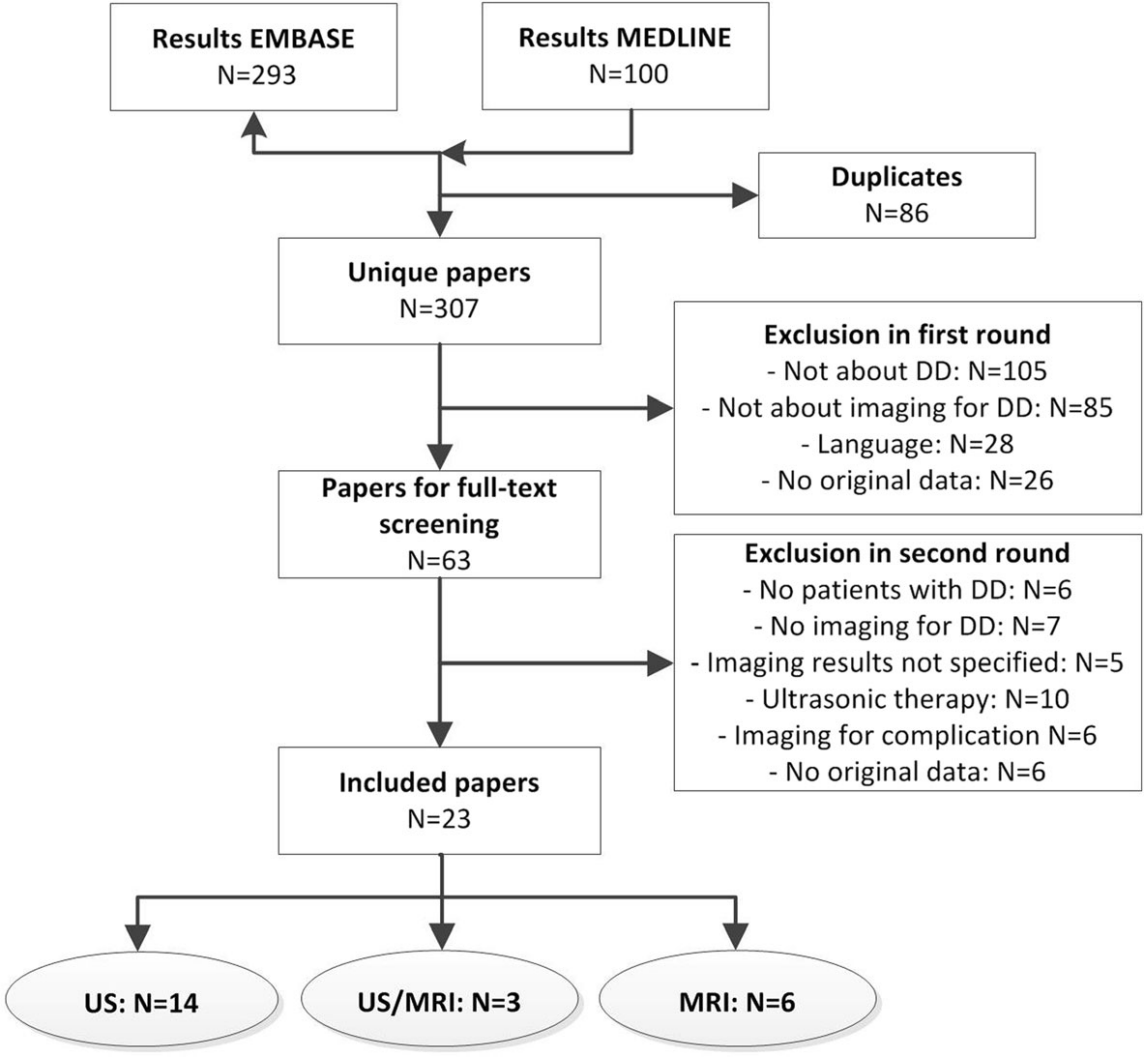
Flow-chart of process of article selection

The initial search yielded 307 unique studies. Of these studies, 244 studies were excluded during the first round. After assessment of the remaining 63 studies in the second round, 23 studies were included in our study. All studies described the use of US and/or MRI. Studies on PET/CT were not found. The process of article selection is shown in Fig. 1.

Five different applications of US and/or MRI for DD patients were identified: diagnosis, measurement of disease extent, measurement of disease activity, guidance of minimally invasive procedures and evaluation of treatment.

### Diagnosis

Seven articles were found that report the use of US and/or MRI for diagnosing DD (Table 3).

**Table 3:** Summary of studies on imaging for diagnosis of DD

Study (year)	Study design	Level of evidence	Imaging modality	N	Clinical details	Outcome measures	Results	Additional value MRI	Additional value US
Juvenspan (2014) [18]	Case-report	5	US + MRI	1	37-year-old female with a mass at the distal end of Guyon’s canal in the right hand	Diagnosis of swelling with unknown origin	No suspected diagnosis following imaging	No	No
Mordus (2017) [19]	Case-report	5	US + MRI	1	64-year-old man with left hand clumsiness and loss of muscle mass between 1st and 2nd rays	Diagnosis of unknown cause of symptoms	No suspected diagnosis following imaging	No	No
Habash (2007) [20]	Case-report	5	MRI	1	36-year-old man with a 2-year history of a steadily enlarging mass of the right volar forearm	Diagnosis of swelling with unknown origin	Suspected fibroplastic sarcoma following imaging	No	–
Kraus (2012) [21]	Case-report	5	US + MRI	1	7-year-old girl with swelling in the left palm (4th ray)	Diagnosis of swelling with unknown origin	Suspected ganglion cyst following imaging	No	No
Spyropoulou (2016) [22]	Case-report	5	MRI	1	10-year-old boy with a nodule and a contracture of the distal interphalangeal joint of the right little finger	Diagnosis of swelling with unknown origin	Suspected fibrous histiocytoma following imaging	No	–
Germano (2016) [23]	Case-report	5	US	1	71-year-old man with clinical signs of Ledderhose, Peyronie and suspected DD and long-term use of primidone for essential tremor	Confirmation of diagnosis	Suspected DD following imaging	–	Yes
Abogamal (2016) [24]	Cross-sectional	4	US	8	Suspected DD patients in a larger study of 114 diabetic patients with or without hand-pain	Confirmation of diagnosis	Suspected DD following imaging	–	Yes

*US* ultrasound, *MRI* magnetic resonance imaging, *DD* Dupuytren disease

Five studies described the use of US and/or MRI for the diagnosis of patients with DD, that had an atypical presentation [18–22]. In two cases, US and MRI did not lead to a diagnosis prior to surgery and DD was diagnosed upon histology [19, 21]. In the other three case-report US and/or MRI led to a wrong diagnosis prior to surgery and histology showed that the final diagnosis was DD [18, 20, 22].

In the two other studies, US was used to diagnose DD in patients with a more typical presentation, in which US was helpful in confirming the diagnosis that was based on clinical examination [23, 24].

In summary, imaging was not instrumental in diagnosing DD in any of the patients with an atypical presentation, but did assist in the diagnosis of DD in patients with a more typical presentation.

### Measurement of disease extent

Two studies described the use of MRI to measure disease extent (Table 4). The first was a case-report in which MRI displayed characteristic features regarding signal intensity and demonstrated the severity and depth of the different fibromatoses (including DD) [25]. According to the authors, MRI is the best imaging modality to delineate the margins and depth of soft-tissue invasion of these lesions and that it can be helpful in guiding appropriate clinical management. The other study was a prospective case-series in which MRI was used to assess 11 hands of 10 patients with DD, that were scheduled for fasciectomy [26]. MRI accurately detected 96% of the cords and 93% of the nodules prospectively, confirmed by surgery and pathology. One cord that was missed, was detected post hoc. One nodule that was missed, was not detected post hoc because it was small and could not be distinguished from a cord. Disease extent corresponded closely to the surgical and gross pathological findings.

**Table 4:** Summary of studies on imaging for measurement of disease extent

Study (year)	Study design	Level of evidence	Imaging modality	N (hands)	Clinical details	Outcome measures	Results	Additional value MRI	Additional value US
English (2012) [25]	Case-report	5	MRI	1	59-year-old woman with Ledderhose disease, knucklepads and DD.	Signal intensity, disease margins and depth	Detailed display of MRI signal intensity and demonstration of severity and depth of the different fibromatoses.	Yes^a^	–
Yacoe (1993) [26]	Prospective case-series	4	MRI	10 (11)	DD patients undergoing fasciectomy	Disease extent on MRI compared by surgery/histology	Accurate detection of 22/23 cords and 13/14 nodules prospectively.	Yes	–

*MRI* magnetic resonance imaging, *DD* Dupuytren disease

^a^ according to authors

These studies show that MRI can accurately assess disease extent. However, for US it is unknown, as there were no available studies on the use of US to measure disease extent.

### Measurement of disease activity

Three studies reported the use of US or MRI to measure disease stage of DD, of which 2 report on findings using US (Table 5). One was a case-report in which Dupuytren tissue was highly vascular and had mixed echogenicity, which were interpreted by the authors as signs of early DD [27]. Furthermore, US-elastography, which can evaluate the elasticity of soft tissues, showed that the thickened aponeurosis and nodules had a firm structure compared to the surrounding tissue. According to the authors, US-elastography could be a potential diagnostic for the differentiation between acute and chronic DD findings. In the second study, a cohort of DD patients, undergoing either enzymatic lysis with CCH-injections or PNF, was followed prospectively [28]. Prior to treatment, the cords of 38 patients were classified with US as either nodular or fibrillar and were assessed for echogenicity (hyper, iso or mixed). Twenty-four (64%) cords had mixed echogenicity and no hypo-echogenic cords were found. After 2 years, 21 (54%) of the patients retained a straight finger, without the formation of a new cord. Fourteen patients (53%) with a nodular cord and 1 patient (17%) with a fibrillary cord had signs of residual or recurrent disease after 2 years. Three patients with signs of residual disease had a recurrent contracture, all of these patients had nodular cords with mixed echogenicity.

**Table 5:** Summary of studies on imaging for measurement of disease activity

Author (year)	Study type	Level of evidence	Imaging modality	N (hands)	Clinical details	Outcome measures	Results	Additional value MRI	Additional value US
Ulusoy (2015) [27]	Case-report	5	US - (elastography)	1	77-year-old male with bilateral contractures of thumb and little fingers.	Echogenicity, vascularity and elasticity.	Thickened palmar fascia with high vascularity and mixed echogenicity Differences in stiffness of DD tissue	–	Yes^a^
Vanek (2018) [28]	Prospective cohort study	3	US	38	DD patients undergoing either PNF or CCH-injections.	- Nodularity + echogenicity of cords - 2-year follow-up for signs of residual disease (palpable cord or recurrent contracture)	Palpable cord: - fibrillar: 1/6, nodular: 14/32 Recurrent contracture: - fibrillar: 0/6, nodular: 3/32	–	Yes
Yacoe (1993) [26]	Prospective case-series.	4	MRI	10 (11)	DD patients undergoing fasciectomy	- MRI signal intensity - Cellularity (histology)	- 22 cords and 3 nodules with low or low to intermediate signal intensity and hypo-cellularity - 10 nodules with intermediate signal and focal areas of high or low signal intensity and high cellularity or mixed composition	Yes	

*US* ultrasound, *MRI* magnetic resonance imaging, *DD* Dupuytren disease, *PNF* percutaneous needle fasciotomy, *CCH* collagenase clostridium hystoliticum

^a^ according to authors

The third study described the use of MRI to measure disease stage by correlating MRI signal intensity to histological results [26]. In total, 22 cords and 13 nodules were found. In all cords and nodules, a low to intermediate signal intensity corresponded to low cellularity and an intermediate to high signal intensity corresponded to high cellularity or mixed composition.

These studies suggest that echogenicity/elasticity and MRI signal intensity are a reflection of disease activity, of which the last two studies have substantiated this hypothesis with study results.

### Guidance of minimally invasive procedures

Seven studies described the use of pre- or peri-operative US for enhancement of safety and improvement of outcomes of minimally invasive procedures (Table 6). Three studies focused on pre-operative detection of displaced neurovascular (NV) bundles using Doppler-US, which focuses on blood flow of the digital artery [29–31]. In two studies, US was used to prospectively detect displaced NV-bundles in several patients with severe Dupuytren contractures undergoing fasciectomy [29, 30]. The surgical findings all corresponded to the US findings in these studies. In the third study, US was used to prospectively detect a displaced NV-bundle in a cohort 48 DD patients undergoing PNF [31]. When a displaced NV-bundle was detected, the site was marked and during the procedure the needle was inserted proximal or distal to the marked site. There was no instance of post-operative neurovascular dysfunction.

**Table 6:** Summary of studies on imaging for pre and peri-operative guidance of minimally invasive procedures

Author (year)	Study type	Level of evidence	Imaging modality	N (fingers)	Clinical details	Outcome parameters	Results	Additional value MRI	Additional value US
Elsahy (1976) [29]	Prospective case-series	4	Pre-operative US	unknown	DD patients undergoing fasciectomy.	Course of NV-bundles	Course of NV-bundle with US corresponded to surgical findings	–	Cannot be determined
Uehara (2012) [30]	Case-control study	4	Pre-operative US	- DD patients: 14 (25) - Healthy volunteers: 22	DD patients, of which 8 underwent fasciectomy, and healthy volunteers.	Sensitivity /specificity of detecting displaced NV-bundle	- Sensitivity/specificity: 80%/76% when difference in depth between ulnar and radial bundle> 3 mm - US-findings corresponded to surgical findings in operated cases.	–	Cannot be determined
Sakellariou (2015) [31]	Prospective cohort study	3	Pre-operative US	48 (90)	DD patients undergoing PNF	- Complications - Immediate correction of contracture - Recurrence at 26 months	- Complications: no tendon rupture or damage to NV-bundle - Correction: MCP 80% PIP 66% - Recurrence (requiring surgery): 18.2%	–	Cannot be determined
Sampson(2011) [32]	Case-report	5	Peri-operativeUS	1	64-year-old woman undergoing PNF and osteopathic manipulative treatment	- Complications’ - Correction of contracture	No complications. Full extension after 5th round of treatment.	–	Cannot be determined
Leclère (2013) [33]	Prospective cohort study	3	Peri-operative US	33 (43)	DD patients undergoing CCH injections	- complications - Correction of contracture: immediate and at 9.9 months - Subjective patient satisfaction - DASH-questionaire	- No tendon rupture or damage to NV-bundle - Immediate correction: MCP 90%, PIP 84% Correction at 9.9 months: MCP 77%, PIP 59% - Satisfaction: 81% - DASH-score: significant decrease during follow-up (*P* < 0.001)	–	Cannot be determined
Croutzet (2017) [34]^a^	Prospective cohort study	4	Peri-operativeUS	(105)	DD patients undergoing minimally invasive procedure	Complications	No instance of tendon rupture or damage to NV-bundle	–	Cannot be determined
Croutzet (2017) [35]^a^	Prospective cohort study	4	Peri-operativeUS	(12)	DD patients undergoing minimally invasive procedure	Hematoma	No instance of specific bleeding or hematoma.	–	Cannot be determined

*US* ultrasound, *MRI* magnetic resonance imaging, *DD* Dupuytren disease, *PNF* percutaneous needle fasciotomy, *CCH* collagenase clostridium hystoliticum, *NV* neurovascular, *MCP* metacarpophalangeal, *PIP* proximal interphalangeal, *DASH* Disabilities of the Arm, Shoulder and Hand

^a^ Data-overlap

Four other studies performed ultrasound guided procedures [32–35]. The first showed the results of a patient undergoing US-guided PNF followed by osteopathic manipulative treatment [32]. The patient did not experience post-operative complications. The second study prospectively followed the results of US-guided CCH injections in a cohort of 33 DD patients [35]. No flexor tendon ruptures or damages to the NV-bundle were reported. In the last two studies, by the same authors, complications of a variety of US guided procedures in the hand were evaluated. These studies have suspected data-overlap, however, since the research question differed and no meta-analysis is conducted with the data, we decided to include both studies. In the first study, 513 procedures in 402 patients were conducted, of which 105 were Dupuytren contractures [34]. No instance of tendon-rupture or damage to the NV-bundle was reported in the whole group. In the other study, 63 US-guided procedures were conducted in 43 patients on anti-coagulants, of which 12 were Dupuytren contractures [33]. The anti-coagulants were not interrupted and local anaesthesia with epinephrine was used. No instance of clinically relevant hematoma was reported.

The conclusion of these articles is that US guided minimally invasive surgery is safe and result are satisfactory. However, none of these studies used a control group, which is why the additional value of US cannot be determined.

### Evaluation of treatment

Five studies reported the use of US or MRI to evaluate different operative and non-operative treatment modalities (Table 7). Three studies used US or MRI to follow-up non-invasive treatment [36–38]. In the first study, US was used to follow-up cross-frictional treatment of a patient with early stage DD, which is a therapy that aims to reduce contracture by stretching the Dupuytren tissue [36]. US imaging was unable to detect any changes to the subcutaneous features of the contractures after 8 weeks of treatment. In the second study, triamcinolone acetonide injections were given in 37 DD patients with 49 hands affected with early stage nodules [37]. The injected nodules were assessed with US for size in the sagittal plane prior to injection and were followed with US for 5 years. A significant decrease in size was detected from pre-injection to 6 months follow-up and to the final follow-up. In the third study, MRI was used to follow-up size and signal intensity of superficial fibromatoses of the hands and feet in patients undergoing electron beam therapy (EBT) [38]. Intensity decreased significantly, which was attributed to progression from the proliferative to the residual stage. Mean volume also decreased significantly. Furthermore, patients with the highest pre-treatment intensity score had the biggest decrease in VAS pain scale.

**Table 7:** Summary of studies on imaging for evaluation of treatment

Study (year)	Design	Level of evidence	Imaging modality	N (hands)	Clinical details	Outcome parameters	Results	Additional value MRI	Additional value US
Christie (2011) [36]	Case-report	5	US	1 (1)	A 42-year-old patient undergoing cross-frictional therapy (8 weeks)	- Visible changes: US + clinical examination - ROM - Symptoms (subjective)	- US: no observed subcutaneous changes - Clinical examination: decreased nodule size, skin wrinkling and contractile bands - Increased ROM - Reduced patient-reported symptoms	–	No
Yin (2016) [37]	Prospective cohort study	3	US	37 (49)	Dupuytren patients undergoing injection of triamcinolone acetonide in nodules. Follow-up: 5 years.	- Nodule size on US - Complications	- nodule size: reduction of 40% at 6 months and 56% at 5 years. - complications: none	–	Yes
Banks (2017) [38]	Retrospective cohort study	4	MRI	6 (8)	Patients with superficial fibromatoses of the hand and feet undergoing EBT Follow-up 4.5 months.	- Nodule volume on MRI - MRI signal intensity - Pain (VAS-score)	- Volume: significant decrease from 1.5 to 1.2 cm^3^ - Signal intensity: significant decrease - Pain: decrease in VAS-score in patients with high pre-treatment signal intensity	Yes	–
Strömberg (2017) [39]	Prospective cohort study	3	US	39	19 patients undergoing CCH, 20 patients undergoing PNF. Follow-up 1 year.	- Gap-width measured with US - Correction of MCP-joint - Recurrence	- Gap-width (median) 18 mm for both groups - MCP-correction (median): PNF 46° and CCH 53° - Recurrence: *n* = 1	Yes	
Crivello (2015) [40]	Prospective cohort study	4	MRI	5 (5)	5 DD patients undergoing CCH in 5 fingers. Follow-up 30 days.	- MRI signal intensity - Cord volume	- MRI signal intensity: significant increase (320%) - Cord volume: significant decrease (72%)	Yes	

*US* ultrasound, *MRI* magnetic resonance imaging, *DD* Dupuytren disease, *PNF* percutaneous needle fasciotomy, *CCH* collagenase clostridium hystoliticum, *EBT* electron-beam therapy, *MCP* metacarpophalangeal, *ROM* range of motion, *VAS* visual analogue scale

Two studies used US or MRI for the follow-up of minimally invasive procedures. In the first study, gap width of ruptured cords was evaluated with US, following CCH or PNF [39]. In all patients undergoing PNF and in 80% of patients undergoing CCH a single gap was detectable at the injection site and there was no significant difference in gap width between the groups. Furthermore, post-operative outcome, with a follow-up of 1 year, was comparable in the two groups. In the second study, MRI was used to evaluate if CCH disrupts or digests the Dupuytren cord [40]. Five patients were examined and MRI showed that signal intensity of the injected cords increased significantly, most likely because of tissue reaction to the injected CCH. Furthermore, the volume of a Dupuytren cord decreased significantly at 30 days post-injection. In summary, US and MRI were both used for the follow-up of different treatment modalities and a variety of outcome parameters was measured following treatment, like volume, signal intensity and gap-width of a cord.

## Discussion

With the current evolution in the knowledge of DD, it is likely that treatment will move towards a more individualized algorithm [8]. Instead of just aiming at reduction of contractures in patients with an advanced stage of the disease, the ultimate goal is to develop a strategy that can distinguish between benign forms of DD, with no or hardly any progression, and more severe forms that do progress. Within the latter group, such a strategy would enable us to differentiate patients that will only need treatment once from the most severe cases that are at risk of rapid progression and recurrence after treatment. Especially for this last category, efforts should be made to create a therapy that prevents progression (eg. anti-inflammatory drugs, anti-mitotic drugs, radiotherapy) [41–44]. With this ongoing evolution in treatment of DD, there is need for reliable instruments that can assess and monitor disease activity and measure disease extent. This is particularly relevant for patients with early stage, active disease that may be eligible for preventive treatment. It is suggested that imaging may be able to play a role here, especially in the evaluation of disease activity, for which no other outcome measure is currently available [11, 26]. This systematic review aimed to investigate the current knowledge of imaging for DD and for what purposes imaging in patients with DD has been used.

Only studies on the use of US and MRI were found and no studies on the use of CT. A variety of applications for the use of US and MRI for DD was found, which could broadly be divided in 5 categories: diagnosis, measurement of disease extent, measurement of disease activity, guidance of minimally invasive procedures and evaluation of treatment.

### Diagnosis

As pointed out in the introduction, DD is usually diagnosed by physical examination [10]. However, in all case-reports that described the use of US and/or MRI for the diagnosis of DD because of an atypical presentation, histology was required to make a final diagnosis, which is the gold standard [18–22]. This implies that US and MRI cannot differentiate DD from other soft tissue diseases to set the diagnosis. However, this can also be a reflection of the lacking knowledge of typical imaging features that characterise DD on US and MRI.

Furthermore, two studies concluded that US may be helpful in supporting the diagnosis for patients with a more typical presentation of DD [23, 24]. However, it is questionable if US is of additional value when clinical signs of the disease are evident.

In our opinion, imaging should still be performed for certain patients, to acquire additional information such as extent, dimensions and affection of neighbouring structures of an undefined lesion, but there is no place for routine imaging in the diagnosis of DD.

### Measurement of disease extent

Two studies point out that MRI can accurately measure disease extent of DD [25, 26], which may be valuable in clinical management. However, at present, the choice of surgery is not primarily based on the extent of the disease, but more on the severity of contracture and patient complaints, which can also be monitored using physical examination [10, 45]. This is why the use of MRI for measurement of disease extent seems to be a cost-ineffective method to add to regular monitoring of patients with DD.

### Measurement of disease stage

Several studies hypothesise that there is a relation between echogenicity and signal intensity of Dupuytren tissue and disease stage [26–28]. If US and MRI are indeed able to reflect cellularity of nodules and cords and hereby disease stage, this would be of importance in the monitoring of patients with early disease.

However, the overall evidence is poor. One study reports on the use of US-elastography and hypothesizes that it may differentiate both the acute and chronic findings in DD [27]. Unfortunately, this study comprised of only one patient and results were not substantiated with histology or follow-up. Another study concluded that echogenicity of Dupuytren cords may be a related to recurrence [28]. However, the inter-rater reliability of assessing nodularity of cords was poor (Cohen’s kappa = 0.38). Also, the authors did not conduct any statistical analyses to show a significant difference in the occurrence of recurrence between fibrillar and nodular cords. Finally, no clear definition of recurrence was used in this article. Recurrence was defined as either residual disease (a palpable cord without recurrent contracture) or recurrent contracture. In our opinion this definition is nonspecific, as DD tissue is not excised during CCH-injections and PNF, which is why it is expected that most patients have signs of residual disease. Recurrent contracture is more clinically relevant, but for this outcome parameter no cut-off value was described. The relation between echogenicity and activity of a DD nodule has also been reported in a descriptive article by Créteur et al. [11]. This article was not included in the analyses since the conclusions were based on an expert opinion of the author and no patient data were provided.

The final study in this category showed that MRI signal intensity corresponds to disease stage, which was determined using the gold standard histology [26]. These results seem promising, however, as US is easier to access, less expensive and patient-friendlier than MRI, it would be very interesting to investigate if echogenicity also corresponds to cellularity in the future. If this is the case, US can be used regularly to assess if patients are at risk of an aggressive course of DD, which is helpful in disease monitoring and in the future also for the selection of patients that are eligible for treatment aiming at disease control [41, 46].

### Guidance of minimally invasive procedures

The main reason to perform US-guided minimally invasive procedures is to enhance safety. In addition to that US-guidance may optimize results. The available literature showed that displaced NV-bundles could be detected using (Doppler)-US [29, 30] and that US-guided minimally invasive surgery had a low complication rate (no incidence of flexor tendon rupture or damage to NV-bundle) [31–35]. Furthermore, ultrasound guided procedures had satisfactory results [31, 32, 35]. However, no study used a control group of patients undergoing non-US-guided minimally invasive surgery. When comparing the results to studies that did not perform US, there does not seem to be much difference in both complication rate and reduction of contracture [47–54]. A randomized controlled trial should be conducted to analyse whether US is really of additional value in pre- and peri-operative management.

### Evaluation of treatment

The last application that was described for US and MRI, was evaluation of several treatment modalities. The number of studies reporting the outcomes of non-surgical treatment aiming at disease control of patients with early DD is increasing [41, 55]. As these patients do not have contractures yet, there is need for an alternative reliable outcome parameter. This is why several studies report the use of imaging to follow-up treatment outcome of non-surgical procedures for patients with early DD [36–38]. In our opinion, the use of US and MRI to follow-up size and signal of early DD nodules is most relevant as, currently, the only other reliable measurement instruments for patients without contractures is physical examination, which only measures area of disease in one plane and measures the projection of DD on the overlying skin [10]. However, no information on the reliability of these imaging modalities for the measurement of area of early DD is available yet. Studies covering the reliability of multiple measurements by a single observer (intra-observer reliability) and measurements by multiple observers (inter-observer reliability) have to be conducted first, to determine the accuracy of US and MRI in the measurement of disease extent in patients with early DD.

Furthermore, imaging for the follow-up of minimally invasive surgery in patients with contractures was described [39, 40]. The results of follow-up of CCH-injections were contradicting. While one study observed an overall decrease of the DD cords [40], the other study observed a local disruption at the injection site comparable to that of PNF [39]. This may be caused by the difference in follow-up time and also by the different imaging modality used (MRI vs US). A study measuring cord volume multiple times following PNF and CCH-treatment could give more insight. However, the relevance of such a study is questionable as there was no difference in surgical outcome and recurrence between PNF and CCH [39], which is supported by previous literature on the outcomes of CCH-injection vs PNF [56].

### Limitations

Generating a clear overview about imaging for DD was challenging, as there was a wide variety of described applications and overall the included studies had a low level of evidence. Ten studies were case-reports, including only 1 patient [18–23, 25, 27, 32, 36]. In three other studies, less than 10 DD patients were included [24, 38, 40] and in one study the number of observed patients was not described [29]. Of the 9 other studies that did describe a larger cohort of DD patients [26, 28, 31, 35, 37, 39], two studies were conference proceedings [33, 34] and only 1 study included a control group with healthy volunteers for a part of the study [30]. All studies were observational, and most lacked adequate statistical methods. The median level of evidence was 4, and no randomized controlled trials were found.

The inclusion of case-reports and conference proceedings may also be seen as weakness of this study. However, as this is the first systematic review on imaging for DD specifically, it was of interest to include as many studies as possible that showed original data, so that the provided information was as complete as possible. Although the search string that was used was selected to be inclusive, it is possible that some studies were not found by our review. Some studies may have used imaging, but not mentioned this in the title, abstract or keywords. However, because of this it is unlikely that these studies aimed to emphasize the value of imaging for DD. Also, we decided to exclude review articles. Although some of these articles did show original US/MRI-images of patients with DD [11–13, 57–67], no original data on one of the possible applications of imaging for DD were given in these articles or the information provided was based on an expert opinion.

Another limitation is that there is a risk of publication bias. Studies that found a valuable application of imaging for DD are more likely to be published than studies that did not show any relevant findings. Finally, relevant articles may have been missed because they were excluded based on language.

## Conclusions

Despite the variety of study designs and overall low level of evidence of the available literature, our review shows that there are interesting applications for imaging in the management of DD patients. The greatest value of imaging seems to lie in the monitoring of disease activity and outcome of non-surgical treatments for patients with early disease. As mentioned in the introduction, treatment of DD patients is currently predominantly aimed at correction of contractures. But when looking at the literature, the focus of research is moving towards the prevention of contractures in patients with early DD and the creation of an individualized treatment algorithm [8, 41, 55]. For the development of treatment aiming at disease control, a reliable outcome measure that can provide information about disease stage and extent in patients with early disease is required. If further research proves that disease activity can be measured with imaging, and with US in particular as it is less expensive and easier to access, it could be a part of the regular monitoring of DD patients. However, before US can be implemented for this purpose, the hypothesis that echogenicity corresponds to cellularity needs to be substantiated by histological results. Also, agreement-studies on the reliability of US for the measurement of early DD have to be conducted.

## Abbreviations

CCH: Collagenase *Clostridium Histolytocum*
CT: Computed tomography
DD: Dupuytren disease
MRI: Magnetic resonance imaging
NV: Neurovascular
PET: Positron emission tomography
PNF: Percutaneous needle fasciotomy
US: Ultrasound
